# An Effective Method to Identify Heritable Components from Multivariate Phenotypes

**DOI:** 10.1371/journal.pone.0144418

**Published:** 2015-12-14

**Authors:** Jiangwen Sun, Henry R. Kranzler, Jinbo Bi

**Affiliations:** 1 Department of Computer Science and Engineering, University of Connecticut, Storrs, Connecticut, United States of America; 2 Treatment Research Center, University of Pennsylvania Perelman School of Medicine and Philadelphia VAMC, Philadelphia, Pennsylvania, United States of America; Fred Hutchinson Cancer Research Center, UNITED STATES

## Abstract

Multivariate phenotypes may be characterized collectively by a variety of low level traits, such as in the diagnosis of a disease that relies on multiple disease indicators. Such multivariate phenotypes are often used in genetic association studies. If highly heritable components of a multivariate phenotype can be identified, it can maximize the likelihood of finding genetic associations. Existing methods for phenotype refinement perform unsupervised cluster analysis on low-level traits and hence do not assess heritability. Existing heritable component analytics either cannot utilize general pedigrees or have to estimate the entire covariance matrix of low-level traits from limited samples, which leads to inaccurate estimates and is often computationally prohibitive. It is also difficult for these methods to exclude fixed effects from other covariates such as age, sex and race, in order to identify truly heritable components. We propose to search for a combination of low-level traits and directly maximize the heritability of this combined trait. A quadratic optimization problem is thus derived where the objective function is formulated by decomposing the traditional maximum likelihood method for estimating the heritability of a quantitative trait. The proposed approach can generate linearly-combined traits of high heritability that has been corrected for the fixed effects of covariates. The effectiveness of the proposed approach is demonstrated in simulations and by a case study of cocaine dependence. Our approach was computationally efficient and derived traits of higher heritability than those by other methods. Additional association analysis with the derived cocaine-use trait identified genetic markers that were replicated in an independent sample, further confirming the utility and advantage of the proposed approach.

## Introduction

Identifying genetic variation that underlies complex phenotypes has important implications for genetics and biology [1, 2]. The power of most gene discovery studies is positively associated with the heritability of the trait [3]. Higher heritability of a trait implies that the trait varies due to stronger genetic influence. Thus, there is greater chance to detect its genetic causative variants. The narrow sense heritability *h* ^2^ is defined by the percentage of phenotypic variance that is due to additive genetic effects. The broad sense heritability *H* ^2^ is defined by the proportion of phenotypic variance due to all genetic variation.

Many complex phenotypes comprise a variety of low level traits (or phenotypic features) that are often highly variable. Association analysis of such a complex phenotype is impeded by this phenotypic heterogeneity [4]. For example, the diagnosis of drug dependence is determined by various patterns of drug use, their effects, and related behaviors [5]. A binary multivariate trait defined by the diagnosis of cocaine dependence, which partitions the population into cases (subjects with the disorder) and controls (subjects without the disorder), cannot differentiate the heterogeneous manifestations of the disease. Because of this, the success of identifying genetic variants is limited when using this binary trait in association analysis [6, 7]. Identifying highly heritable components of the disease could permit the detection of genetic variants that are not detectable using the standard diagnosis-based traits [8–12]. Efforts have been made to enhance the binary trait by capturing more phenotypic variation, such as defining a multivariate trait as symptom count [7]. However, this kind of multivariate trait can have low heritability and may thus be sub-optimal for association analysis.

Heritable component analysis methods identify principal components of the data, i.e., linear combinations of low level traits, that are heritable [13–16]. All current methods decompose the identification of heritable components into solving two separate subproblems in sequence. They first estimate two covariance matrices of the low-level traits: **Σ**_*a*_, the variance due to additive genetic effects taking into account the relationships of individuals (family structure); and **Σ**, the covariance matrix due to effects other than additive genetic effects. If there are *d* low level traits in **x**, this means that two *d*-by-*d* matrices need to be estimated from the sample. Once the two covariance matrices are computed, a generalized eigenproblem is solved to identify the combination coefficients **w** so that the ratio of **w** ^⊤^ **Σ**_*a*_ **w**/**w** ^⊤^ **Σ** **w** is maximized, leading to high heritability for the combined trait **w** ^⊤^ **x**.

A few methods have been developed in the literature to estimate the two covariance matrices. In [14, 15], the two matrices are estimated based on the genetic effect of a single quantitative-trait locus to all the low level traits. This method has limited utility when the variance-covariance of the low level traits is due to multiple genetic loci (which is often the case for complex phenotypes). In [13, 16, 17], the two covariance matrices are estimated from family pedigrees of the sample. The approach used in [13] takes only siblings in a family, so it is inadequate to handle general (complex) pedigrees. The two approaches in [16] and [17] can handle general pedigrees. The first one derives an analytic formula for the covariance matrices based on Analysis of Variance (ANOVA). Although reducing the computational cost, the analytic formula is unable to take into account the fixed effects from covariates such as sex, age or race, which is also a problem for the method in [13]. Currently, the most comprehensive approach is a maximum likelihood method [17] that can estimate the fixed effects and covariance matrices together, but this method is computationally prohibitive as discussed in [16]. Even when *d* = 20 low level traits are used, this method can run for days, and as observed in our experiments, the method may not converge. It requires very large sample to obtain reliable estimates of two covariance matrices and *d* combination coefficients, totally 2*d* ^2^ + *d* parameters, from a sample.

We show that, to obtain highly heritable components of a multivariate trait, the estimation of two covariance matrices is unnecessary. We propose an optimization approach that directly identifies a linear combination of low level traits whose estimated heritability is maximized. This optimization problem is formulated by decomposing the maximum likelihood method for estimating trait heritability. An *sequential quadratic programming* algorithm is developed to optimize the problem. We then extend the basic formulation to correct fixed effects of covariates in the component analysis. Because we do not estimate any covariance matrix, our approach is computationally much more efficient than those in [13, 17]. The proposed approach is validated in both simulations and a case study on cocaine dependence. The effectiveness of the approach is demonstrated not only by the higher cross-validated heritability of the derived traits than the existing methods but also by a follow-up association study that compares the utility of the derived traits with the commonly used phenotype. Specifically, a highly heritable multivariate trait was derived for cocaine dependence. More statistically significant associations were found for this trait than for a symptom-count phenotype.

## Methods

We first introduce the standard methods for heritability estimation, and then derive our formulation that maximizes the heritability of a linearly-combined trait. An efficient algorithm is developed to optimize the formulation. At last, we extend the approach to take into consideration the fixed effects of covariates.

### Background: Heritability Estimation

To estimate the heritability of a quantitative trait *y*, the well established maximum likelihood method is based on linear mixture models [3, 18]. The method assumes that the phenotype **y** ^*i*^ of a family *i* follows a multivariate normal distribution with covariance **Ω**_*i*_ and separate means for male and female family members, *μ*_*m*_ and *μ*_*f*_, respectively. Separate means are used for males and females based on the general observation that males and females present differences in quantitative traits, such as height and weight. The (*j*, *k*)-th entry of **Ω**_*i*_ is the phenotypic covariance of two family members *j* and *k*, given by

$$cov\left(y_{j}^{i},y_{k}^{i}\right)=2\sigma_{a}^{2}\Phi_{jk}^{i}+\sigma_{d}^{2}\Delta_{jk}^{i}+\sigma_{e}^{2}\Gamma_{jk}^{i}$$ (1)

where $\sigma_{a}^{2}$ and $\sigma_{d}^{2}$ are the variance components due to additive and dominant genetic effects, respectively, and $\sigma_{e}^{2}$ denotes the variance component due to environmental factors. Eq (1) can be extended to include other effects, such as an epistatic genetic effect $\sigma_{I}^{2}$. The quantity $\Phi_{jk}^{i}$ is the kinship coefficient between members *j* and *k*. It is the probability that two alleles randomly drawn from *j* and *k* at a genetic locus are identical by descent (IBD), i.e., that these two alleles are identical copies of the same ancestral allele. An allele is one of the alternative forms at a genetic locus. As the human genome is diploid, each individual has two copies of an allele that may differ at a genetic locus. The quantity $\Delta_{jk}^{i}$ is the probability that members *j* and *k* share both alleles at a genetic locus. Both matrices **Φ**_*i*_ and **Δ**_*i*_ can be calculated from the family pedigrees [3]. Example entries of **Φ** and **Δ** between selected family members are illustrated in Table 1 where random mating is assumed. The parameter $\Gamma_{jk}^{i}$ is an environmental indicator that encodes whether *j* and *k* live together ($\Gamma_{jk}^{i}=1$) or apart ($\Gamma_{jk}^{i}=0$).

**Table 1:** Elements of the matrices Φ and Δ for selected relationships in a family when random mating is assumed.

Relationship	Φ	Δ
Same person	1/2	1
Parent-Child	1/4	0
Full-siblings	1/4	1/4
Half-siblings	1/8	0
Monozygotic twins	1/2	1
Grandparent-grandchild	1/8	0
Uncle/aunt-nephew/niece	1/8	0
First cousins	1/16	0
Double first cousins	1/8	1/16
Spouses	0	0

The narrow sense heritability is given by $h^{2}=\sigma_{a}^{2}/\sigma_{p}^{2}$ where $\sigma_{p}^{2}$ is the total variance in *y*, i.e., $\sigma_{p}^{2}=\sigma_{a}^{2}+\sigma_{d}^{2}+\sigma_{e}^{2}$, while the broad sense heritability is given by $H^{2}=\left(\sigma_{a}^{2}+\sigma_{d}^{2}\right)/\sigma_{p}^{2}$. In this paper, we target at quantitative traits with higher narrow sense heritability, which we henceforth simply refer to as heritability. However, our formulation can be easily modified to derive a quantitative trait of high *H* ^2^.

The five parameters, *μ*_*m*_, *μ*_*f*_, $\sigma_{a}^{2}$, $\sigma_{d}^{2}$ and $\sigma_{e}^{2}$, are estimated by maximizing the log likelihood of the trait values over all sample families [18]. The log likelihood is computed by

$$LL=\sum_{i}-\frac{1}{2}\ln\left|\Omega_{i}\right|-\frac{1}{2}{\left(y^{i}-\mu^{i}\right)}^{⊤}\Omega_{i}^{-1}\left(y^{i}-\mu^{i}\right),$$ (2)

where *μ* ^*i*^ denotes a vector of the means *μ*_*m*_ and *μ*_*f*_ for male or female members, respectively, in the family *i*. The gradient and Hessian of Eq (2) with respect to *μ*_*m*_, *μ*_*f*_, $\sigma_{a}^{2}$, $\sigma_{d}^{2}$ and $\sigma_{e}^{2}$ can be calculated, and a Newton-Raphson algorithm or a scoring method [18] can be applied to maximize the log likelihood Eq (2).

The heritability of a quantitative trait *y* is often estimated with correction for the effects of covariates **z**, such as age, sex, or race. These covariate effects are modeled as fixed effects on *y*. Thus, a linear regression model *y* = **z** ^⊤^ **v** + *ϵ* can be built where **v** indicates the combination weights for the covariates. The heritability of the residual *ϵ* is then estimated using the described maximum likelihood method and treated as the heritability of *y* after adjusting for covariate effects.

### Proposed Quadratic Optimization

In heritability estimation, a trait is given, and we search for the values of $\sigma_{a}^{2}$, $\sigma_{d}^{2}$ and $\sigma_{e}^{2}$ that maximize the likelihood of observing the trait values and compute the heritability as $\sigma_{a}^{2}/\left(\sigma_{a}^{2}+\sigma_{d}^{2}+\sigma_{e}^{2}\right)$. In our study, we solve the inverse problem that a trait must be derived so that its heritability is maximized when estimated by the above maximum likelihood method.

For a given set of *d* phenotypic features **x**, we find a linearly combined trait *y* : *y* = **x** ^⊤^ **w**. If a trait *y* has the highest possible heritability, the covariance of *y* among any family members in family *i*, $cov(y_{j}^{i},y_{k}^{i})$, should be due to the additive effect $\sigma_{a}^{2}$ only, and $\sigma_{d}^{2}=\sigma_{e}^{2}=0$. In other words, for such a trait, the covariance matrix of the phenotype **y** ^*i*^ of a family *i* relies only on the additive effect parameter $\sigma_{a}^{2}$ and the kinship matrix **Φ** ^*i*^, i.e., $\Omega_{i}=2\sigma_{a}^{2}\Phi^{i}$. Thus $\sigma_{a}^{2}$ is equal to the total variance $\sigma_{p}^{2}$ of *y*. We need to search for the values of **w** that maximize the likelihood of observing $\sigma_{d}^{2}=\sigma_{e}^{2}=0$, or in other words, that maximize the likelihood of $\Omega_{i}=2\sigma_{a}^{2}\Phi^{i}$.

Let **X**_*i*_ be the data matrix on the *d* features (as columns) for the subjects (as rows) in family *i*. Then the trait values of the family members form a vector $y^{i}=X_{i}^{⊤}w$. Because *y* is homogeneously dependent on the unknown **w**, **w** can be scaled so that the sample variance of *y* is 1, which implies that $\sigma_{p}^{2}=1$ (and hence $\sigma_{a}^{2}=1$). Substituting the values of **Ω**_*i*_, **y** ^*i*^ and $\sigma_{a}^{2}$ into the log likelihood in Eq (2) yields the following maximization problem:

$$\max_{w,\mu_{m},\mu_{f}}\sum_{i}-\frac{1}{2}\ln\left|2\Phi_{i}\right|-\frac{1}{4}{\left(X_{i}^{⊤}w-\mu^{i}\right)}^{⊤}\Phi_{i}^{-1}\left(X_{i}^{⊤}w-\mu^{i}\right),$$

which is equivalent to the following minimization problem after eliminating constants (for example, $\frac{1}{2}\ln|2\Phi_{i}|$ does not vary in terms of **w**, *μ*_*m*_ or *μ*_*f*_, and thus is a constant,)

$$\min_{w,\mu_{m},\mu_{f}}\sum_{i}{\left(X_{i}^{⊤}w-\mu^{i}\right)}^{⊤}\Phi_{i}^{-1}\left(X_{i}^{⊤}w-\mu^{i}\right).$$ (3)

We then consolidate the parameters **w**, *μ*_*m*_ and *μ*_*f*_ into a single column vector **β** = [**w** ^⊤^, *μ*_*m*_, *μ*_*f*_]^⊤^. Note that *μ* ^*i*^ is a vector of length of the number of family members with corresponding entries equal to either *μ*_*m*_ or *μ*_*f*_ depending on the gender of the family member. We can simplify Eq (4) to have $X_{i}^{⊤}w-\mu^{i}=H_{i}^{⊤}\beta$ and **H**_*i*_ is defined by

$$H_{i}={\left[X_{i}^{⊤},{\left[-1/0\right]}_{i}^{m},{\left[-1/0\right]}_{i}^{f}\right]}^{⊤}$$

where ${\left[-1/0\right]}_{i}^{m}$ and ${\left[-1/0\right]}_{i}^{f}$ are column vectors with length equal to the number of members in family *i*. For males in the family, −1 is assigned at their corresponding entries in ${\left[-1/0\right]}_{i}^{m}$ and 0 at other positions of the vector. The vector of ${\left[-1/0\right]}_{i}^{f}$ is similarly defined for female family members. For instance, a family *i* has three members included in a study, and they are ordered as a male member, a female member and then another male member. The vector ${\left[-1/0\right]}_{i}^{m}={\left[-1,0,-1\right]}^{⊤}$ and the vector ${\left[-1/0\right]}_{i}^{f}={\left[0,-1,0\right]}^{⊤}$, which ensures that ${\left[-1/0\right]}_{i}^{m}\mu_{m}+{\left[-1/0\right]}_{i}^{f}\mu_{f}=-\mu^{i}$. Then, the objective function of Eq (4) becomes

$$\sum_{i}{\left(X_{i}^{⊤}w-\mu^{i}\right)}^{⊤}\Phi_{i}^{-1}\left(X_{i}^{⊤}w-\mu^{i}\right)=\sum_{i}\left(\beta^{⊤}H_{i}\right)\Phi_{i}^{-1}\left(H_{i}^{⊤}\beta \right)=\beta^{⊤}\left(\sum_{i}H_{i}\Phi_{i}^{-1}H_{i}^{⊤}\right)\beta .$$

By stacking the **H**_*i*_ matrices of different families in columns, we get another matrix **H**. Similarly, we can form a matrix **X**, so the trait values of all subjects **y** = **X** ^⊤^ **w**. The sample variance of the trait *y* is, by definition, (1/*n*)(**y** − ***μ***)^⊤^(**y** − ***μ***). It is equal to (1/*n*)(**X** ^⊤^ **w** − ***μ***)^⊤^(**X** ^⊤^ **w** − ***μ***) = (1/*n*)***β*** ^⊤^ **H** **H** ^⊤^ ***β***. Then, the condition of $\sigma_{p}^{2}=1$ corresponds to a constraint on ***β***: (1/*n*)***β*** ^⊤^ **H** **H** ^⊤^ ***β*** = 1, which can be rewritten as **β** ^⊤^ **H** **H** ^⊤^ ***β*** − *n* = 0.

As a matter of fact, *μ*_*m*_ and *μ*_*f*_ are not free parameters, as they are determined once **w** is determined. They are equal to the sample means of the trait, i.e., Mean(**x** ^⊤^ **w**), for male and female, respectively. Let **x**_*m*_ and **x**_*f*_ be the two means of the data vector **x** respectively over male and female samples. Then, $x_{m}^{⊤}w=\mu_{m}$ and $x_{f}^{⊤}w=\mu_{f}$. These equations give two additional constraints. Let $a_{m}={\left[x_{m}^{⊤},-1,0\right]}^{⊤}$, $a_{f}={\left[x_{f}^{⊤},0,-1\right]}^{⊤}$, then the two constraints on ***β*** state that $a_{m}^{⊤}\beta =0$ and $a_{f}^{⊤}\beta =0$.

Imposing all of these constraints on Eq (4) yields an optimization problem where a quadratic objective needs to be minimized subject to a quadratic constraint and two linear equality constraints as follows:

minβaaβ⊤(∑iHiΦi-1Hi⊤)β,subjecttoaaβ⊤HH⊤β-n=0,am⊤β=0,af⊤β=0. (4)

According to statistical learning theory [19], optimizing only the empirical heritability on the training sample as in Eq (4) will lead to the so-called overfitting problem, which means that the resultant model *y* = **x** ^⊤^ **w** has low validation heritability despite a high training heritability. To enhance the generalizability of the derived model to new samples, a regularization condition on **w**, *R*(**w**), is required to control the complexity of the model. The objective function in Eq (4) thus becomes

$$\begin{array}{c} \beta^{⊤}\left(\sum_{i}H_{i}\Phi_{i}^{-1}H_{i}^{⊤}\right)\beta +\lambda R\left(w\right), \end{array}$$ (5)

where *λ* is a pre-specified tuning parameter for balancing the two terms in the objective function, and *R*(**w**) can be realized in different forms and be application-specific. For example, *R*(**w**) can be implemented with the ℓ_1_ vector norm: ${\left||w|\right|}_{1}=\sum_{j=1}^{d}\left|w_{j}\right|$, which is known to create shrinkage effects on **w** as shown in the Least Absolute Shrinkage and Selection Operator (LASSO) method [20]. When features in **x** are clustered in multiple groups and sparsity in the level of each feature group is desirable, *R*(**w**) can be implemented by the ℓ_2,1_ vector norm as used in the group LASSO [21] and defined by ${\left||w|\right|}_{2,1}=\sum_{\ell =1}^{L}\sqrt{\sum_{j\in G_{\ell }}w_{j}^{2}}$ where $G_{\ell }$ contains the indices of the features in the group ℓ.

Specifically, we develop an algorithm in the next section to solve the following optimization problem with the ℓ_1_ norm regularization condition

minβaaβ⊤(∑iHiΦi-1Hi⊤)β+λ||w||1,subjecttoaaβ⊤HH⊤β-n=0,am⊤β=0,af⊤β=0. (6)

Note that Problem Eq (4) is a special case of Problem Eq (6) when *λ* = 0. Hence, a solver for Problem Eq (6) can also solve Problem Eq (4).

### Solving the Proposed Optimization Problem

The objective function in Eq (6) is not differentiable because of the ℓ_1_ norm regularization condition. However, by a widely used change-of-variables strategy, we can convert it into an equivalent differentiable form so gradient based solvers can be used. We introduce two sets of variables **u** ≥ 0 and **v** ≥ 0 both of length equal to that of **w**. We set **w** = **u** − **v**, which gives $X_{i}^{⊤}w=X_{i}^{⊤}u-X_{i}^{⊤}v$. Correspondingly, we replace the parameter vector ***β*** by **γ** = [**u** ^⊤^,**v** ^⊤^, *μ*_*m*_, *μ*_*f*_]^⊤^, and replace **H**_*i*_ by

$$K_{i}={\left[X_{i}^{⊤},-X_{i}^{⊤},{\left[-1/0\right]}_{im},{\left[-1/0\right]}_{if}\right]}^{⊤},$$

so we have **H**_*i*_ ***β*** = **K**_*i*_ ***γ***.

Stacking all **K**_*i*_’s in columns leads to the full matrix **K**. The quadratic constraint in Eq (6) then becomes **γ** ^⊤^ **K** **K** ^⊤^ **γ** − *n* = 0. By setting $b_{m}^{⊤}={\left[x_{m},-x_{m},-1,0\right]}^{⊤}$, $b_{f}^{⊤}={\left[x_{f},-x_{f},0,-1\right]}^{⊤}$, the linear constraints in Eq (6) become $b_{m}^{⊤}\gamma =0$ and $b_{f}^{⊤}\gamma =0$. We have bound constraints on the new variables, i.e., **u** ≥ 0 and **v** ≥ 0. We hence design a matrix **J** = [**I**_2*d* × 2*d*_, [0]_2*d*_, [0]_2*d*_] where **I**_2*d* × 2*d*_ is the identity matrix of dimension 2*d* × 2*d*, and [0]_2*d*_ is a column vector of all zero entries with length of 2*d*. Then, the bound constraints can be written as **J** **γ** ≥ 0. Overall, with the new variables **u** and **v**, Eq (6) can be re-written as follows

minγaaf:γ⊤(∑iKiΦi-1Ki⊤)γ+λ∑j=12dγjsubjecttoaag1:γ⊤KK⊤γ-n=0,g2:bm⊤γ=0,g3:bf⊤γ=0,g4:e:Jγ≥0, (7)

where *e* = 2*d* + 3 is the total number of constraints in the problem.

It can be proved mathematically that the optimal solution of Eq (7) is identical to the optimal solution of Eq (6) in the sense that the optimal **w** = **u** − **v**. Note that the regularization condition in Eq (7), $\sum_{j=1}^{2d}\gamma_{j}$, is just equal to $\sum_{j=1}^{d}\left(u_{j}+v_{j}\right)$. At optimality of Eq (7), either *u*_*j*_ = 0 or *v*_*j*_ = 0 for the *j*th feature because otherwise they would not be optimal. If both *u*_*j*_ > 0 and *v*_*j*_ > 0 and assume *u*_*j*_ ≥ *v*_*j*_, then we have another solution, (${\tilde{u}}_{j}=u_{j}-v_{j}$, ${\tilde{v}}_{j}=0$), that achieves lower objective value than (*u*_*j*_, *v*_*j*_) because the first term of *f* remains the same whereas the second term of *f* is reduced by 2*v*_*j*_. Thus, at optimality, the regularizer of Eq (7) $\sum_{j=1}^{2d}\gamma_{j}=\sum_{j=1}^{d}\left(u_{j}+v_{j}\right)=\sum_{j=1}^{d}|u_{j}-v_{j}|=\sum_{j=1}^{d}\left|w_{j}\right|$.

Although Eq (7) is not a convex problem due to the quadratic equality constraint *g*_1_, we can solve it efficiently by the framework of sequential quadratic programming (SQP) [22]. A SQP algorithm solves the optimization problem iteratively. At each iteration, it approximates the original problem by a convex quadratic program, for which a solution can be easily computed. A quadratic program is defined as a minimization of a quadratic objective function subject to linear constraints. To form the approximate subproblem, the Lagrangian function of Eq (7) is used:

$$L\left(\gamma ,\alpha \right)=f\left(\gamma \right)-\sum_{k}\alpha_{k}g_{k}\left(\gamma \right)$$ (8)

where **α** contains all Lagrange multipliers and *k* indexes the constraints. We use the second-order Taylor expansion to approximate the Lagrangian which forms the quadratic objective function of the subproblem, and use the first-order expansions to approximate the original constraints which form linear constraints for the subproblem.

The gradients of the objective function *f* and the constraints *g*_*i*:*i* = 1:*e*_ with respect to **γ** can be calculated as follows:

$$\begin{array}{c} \nabla f=2(\sum_{i}K_{i}\Phi_{i}^{-1}K_{i}^{⊤})\gamma +\lambda c,\\ \nabla g_{1}=2\left(KK^{⊤}\right)\gamma ,\;\;\nabla g_{2}=b_{m},\\ \nabla g_{3}=b_{f},\;\;\nabla g_{4:e}=c \end{array}$$

where $c={\left[{\left[1\right]}_{2d}^{⊤},0,0\right]}^{⊤}$ and [1]_2*d*_ is a column vector of all ones with length of 2*d*. The Hessian of L with respect to ***γ*** is calculated as:

$$\nabla_{\gamma \gamma }^{2}L=2\sum_{i}K_{i}\Phi_{i}^{-1}K_{i}^{⊤}-2\alpha_{1}KK^{⊤}.$$ (9)

The subproblem at each iteration is formulated based on the current iterates **γ**_*t*_ and Lagrange multipliers **α**_*t*_. At the iteration *t* + 1, the search directions for both **γ** and **α** can be computed by solving the following quadratic program

minpaaf(γt)+∇f(γt)⊤p+12p⊤∇γγ2L(γt,αt)psubjecttoaa∇gk(γt)⊤p+gk(γt)=0,k∈[1:3]∇gk(γt)⊤p+gk(γt)≽0,k∈[4:e] (10)

where **p** is the search direction of **γ**, along which the objective function *f* can be decreased. Let ${\hat{p}}_{t}$ be the solution to this subproblem and ${\hat{q}}_{t}$ be the corresponding optimal Lagrange multipliers of ${\hat{p}}_{t}$, the search direction of **α** is calculated as ${\hat{q}}_{t}-\alpha_{t}$. Then, a line search method, such as those described in [22], can be used to determine a step size of moving along the directions. Then, **γ** and **α** are updated as follows:

$$\gamma_{t+1}=\gamma_{t}+s{\hat{p}}_{t},\;\;\;\alpha_{t+1}=\alpha_{t}+s\left({\hat{q}}_{t}-\alpha_{t}\right).$$ (11)

Algorithm 1 summarizes the SQP algorithm that we developed to solve Eq (7), and hence Eq (6).

**Algorithm 1** A sequential quadratic programming approach to solving Eq (7)

**Input: K**_*i*_, **Φ**_*i*_, $a_{m}^{\prime }$, $a_{f}^{\prime }$, *λ*

**Output: γ**

 1. Initialize **γ** with **u** = **1**, **v** = **0**, and *μ*_*m*_, *μ*_*f*_ equal to the sample male and female means of the obtained trait when **w** = **1**.

 2. Initialize **α** with **α** = **1**.

 3. Evaluate *f*, ∇f, $\nabla g_{k}$ and $\nabla_{\gamma \gamma }^{2}L$ with the current **γ** and **α**.

 4. Solve Eq (10) to obtain $\hat{p}$ and $\hat{q}$.

 5. Perform line search to find the learning step size *s*.

 6. Update **γ** and **α** as in Eq (11).

 Repeat 3–6 until **γ** reaches a fixed point.

### Correction for Covariates

As discussed in the background section, the heritability of a quantitative trait *y* with effects from covariates **z** is equal to the heritability of the residual *ϵ* of the linear model *y* = **z** ^⊤^ **v** + *ϵ*. Therefore, our objective here is to find $\hat{w}$ and $\hat{v}$ that optimize the heritability estimate of *ϵ*: *ϵ* = **x** ^⊤^ **w** − **z** ^⊤^ **v**, as *y* = **x** ^⊤^ **w**. Let **Z**_*p* × *n*_ be the data matrix on **z** of length *p* for the *n* subjects, the residual is calculated for all the subjects as ***ϵ*** = **X** ^⊤^ **w** − **Z** ^⊤^ **v**.

Given the data **Z** and **y**, a linear regression model *y* = **z** ^⊤^ **v** + *ϵ* is typically obtained through a least squares method which has an analytical solution, $\hat{v}={\left(ZZ^{⊤}\right)}^{-1}Zy$. As **y** = **X** ^⊤^ **w**, we have $\hat{v}={\left(ZZ^{⊤}\right)}^{-1}ZX^{⊤}w$ and

$$\epsilon =(X^{⊤}-Z^{⊤}{\left(ZZ^{⊤}\right)}^{-1}ZX^{⊤})w.$$

Let **M** = (**X** ^⊤^ − **Z** ^⊤^(**Z** **Z** ^⊤^)^−1^ **Z** **X** ^⊤^)^⊤^, which can be pre-calculated from data, the calculation of ***ϵ*** can be rewritten as ***ϵ*** = **M** ^⊤^ **w**. Then, the objective of optimizing the heritability of *ϵ* can be translated to finding the optimal **w** that gives an ***ϵ*** of highest estimate of heritability. Comparing to the problem of finding **w** that gives a trait *y* = **x** ^⊤^ **w** with highest possible heritability, the only difference we have here is that the design matrix has been changed from **X** to **M** for the parameters **w**. Therefore, we can use the same SQP algorithm (Algorithm 1) to find the **w** that optimizes the heritability of *ϵ*. An interesting observation in our derivation is that correcting a quantitative trait to account for covariant effects is equivalent to correcting the data matrix that used to derive the trait.

### Algorithm Evaluation

The proposed approach was first validated in simulations where we compared it with the current two-step approaches, i.e., estimating the two covariance matrices from pedigrees first and then solving an eigenproblem. We compared with all the three different methods that can be used to estimate the variance-covariance matrices, which were referred to, respectively, as Ott [13], Anova [16] and ML [17]. The following *Results* section provides the details of the simulation and empirical evidence showing the superior performance of our algorithm.

After validated in simulations, the proposed approach was then used in a case study to analyze a real-world dataset that was aggregated from genetic studies of cocaine dependence (CD) [7, 23]. Our algorithm was able to derive a quantitative trait with higher heritability than that of commonly used CD phenotypes. To show how our approach could help genetic association analysis, we compared the utility of the derived trait against the symptom-count phenotype as traits in association analysis and replicated the findings on a separate sample. The narrow sense heritability of all of the tested traits in this study was estimated by the widely-used *polygenic* function in the Sequential Oligogenic Linkage Analysis Routines (SOLAR) program [24].

#### Ethics statement

The *Semi-Structured Assessment for Drug Dependence and Alcoholism* (SSADDA) dataset [7] was used in both our simulations and the case study to evaluate the proposed algorithm. The SSADDA subjects were recruited from multiple sites, including the University of Connecticut Health Center, Yale University School of Medicine, the University of Pennsylvania School of Medicine, McLean Hospital and the Medical University of South Carolina. All subjects gave written, informed consent to participate, using procedures approved by the institutional review board (IRB) at each participating site. Readers can consult with [7] for the details of subject recruitment in those studies. The SSADDA data were de-identified and the analyses in this present study were approved by the University of Connecticut IRB Protocol H15-045 and the University of Pennsylvania IRB Protocals 804787 and 812856.

## Results

This section provides the details of the simulation process and the case study of CD together with the empirical evidence showing the superior performance of our approach.

### Simulations

In order to make our synthetic data more realistic but with known patterns, we created the synthetic data based on family structures in the SSADDA dataset. In this dataset, there were totally 6810 subjects, of which 1915 were from small nuclear families and the remaining subjects were unrelated individuals. Based on the family structures in this data, we synthesized two quantitative traits following the same assumptions used in the maximum likelihood method for heritability estimation [18].

#### Experimental data and procedure

The values of the first trait *y*_1_ were randomly drawn for each family from a multivariate Gaussian distribution: *N*(***μ***, **Ω**), where ***μ*** and **Ω** were determined as follows. The dimension of ***μ*** was determined by the number of subjects in the family, such that each dimension corresponded to an individual in the family. The value of ***μ*** used in the simulations may vary between families according to the gender of the members. Precisely, if a family member is male, *μ* was set to *μ*_*m*_; otherwise it was set to *μ*_*f*_. The covariance matrix **Ω** was given by the following equation:

$$\Omega =2\sigma_{a}^{2}\Phi +\sigma_{d}^{2}\Delta +\sigma_{e}^{2}I,$$ (12)

where **Φ** and **Δ** were composed according to Table 1. Without loss of generality, in this study we used identity matrix **I** as the matrix **Γ** in Eq (1). The quantitative trait *y*_1_ was simulated with the following choices of the parameters:

$$\left[\sigma_{a}^{2},\;\sigma_{d}^{2},\;\sigma_{e}^{2},\;\mu_{m},\;\mu_{f}\right]=\left[0.8,\;0.1,\;0.1,\;0.9,\;0.3\right].$$ (13)

Hence, 80% of the phenotypic variance was due to additive genetic effects, and the ideal heritability is 0.8 according to Eq (13). By the random nature of the simulation, the actual heritability of the simulated trait may vary a little.

In order to evaluate if our approach can correct for fixed effects of covariates, we created another quantitative trait *y*_2_ by adding effects from age and race to *y*_1_. Let *c*_1_ and *c*_2_ measure the effects of age and race respectively on *y*_2_, we calculated *y*_2_ as follows: *y*_2_ = *y*_1_ + *c*_1_ × *age* + *c*_2_ × *race*. The values of the two *c*’s were arbitrarily set to *c*_1_ = 1.1 and *c*_2_ = 0.7, (which can certainly be set to any other non-zero values). Using SOLAR, we estimated the heritability of *y*_1_ with sex as covariate (*h* ^2^ = 0.796) and the heritability of *y*_2_ with sex, age, race as covariates (*h* ^2^ = 0.797).

We next simulated data of phenotypic features for the two quantitative traits. For each trait, we synthesized a dataset consisting of *d* = 10 relevant phenotypic features. We first specified the weights **w** of the features; then we generated data for these features as follows. For each subject, we randomly picked *d* − 1 features and drew their values randomly from the standard multivariate Gaussian distribution. Assume that the *k*-th feature is the remaining feature. Its value for subject *i* was computed by $\left(y^{i}-\sum_{j=1,j\neq k}^{d}w_{j}x_{j}^{i}\right)/w_{k}$ (where *w*_*k*_ ≠ 0 because these 10 features were created with non-zero weights in the linear model). This procedure guaranteed that the trait $y=\sum_{j=1}^{d}w_{j}x_{j}$, and because the feature computed from the values of other features varied randomly among subjects, every feature had a portion of randomly-drawn data.

In practice, a multivariate trait may not depend on all of the considered phenotypic features. In order to test if our approach can identify the relevant features, we created four other datasets for each of the traits, respectively, consisting of *d* = 20, 30, 40 and 50 features where only the first 10 of them were created following the above procedure, thus relevant to the simulated traits. The other features were all randomly drawn from standard Gaussian distribution and assigned a weight of 0. By simulating the data in this way, there was at least one linear combination of the features in each dataset that led to the simulated traits of high heritability. If our approach is to work, it should find this linear combination which is considered as the groundtruth model. There is a likelihood that another linear combination could give even higher heritability due to the random nature of the data, but this likelihood is small. In our experiments, none of the algorithms could locate any other combinations with higher heritability than the implanted one.

In practice, a multivariate trait may also depend on some features that are not observed. In our simulations, it implied that some of the ten relevant features might be absent. Therein, we further explored how our approach performed in the situation where the data was incomplete by randomly removing relevant features. We experimented with removing one to five relevant features incrementally. Note that in this sensitivity test, there was no longer a groundtruth model for the algorithm to test against because the implanted linear model had been broken with missing features. In this case, if our approach is to work, it should find a combination that leads to a heritability estimate no lower than that of the original features and that derived by other known methods.

The three previous methods evaluated in our comparison all used a regularization condition in their eigenproblem, so they also had a tuning parameter *λ*. In the experiments with each dataset, the parameters *λ* of all methods were tuned in the same three-fold cross validation process. More specifically, for each dataset, we randomly split the sample into three groups, and each group had the same amount of unrelated individuals and families with multiple members whenever it was possible. Samples in each group were used in one of the three folds, respectively, as the validation data to test the heritability of the trait derived by a method from the rest of the samples. We repeated this three-fold cross validation with 10 random splits for each choice of *λ* on each dataset. The choices of *λ* were pre-specified to the range of [0, 50] with a step size of 1. For each method, the choice of *λ* that gave the best cross validated heritability was used in the subsequent analysis.

In the experiments with the trait *y*_1_, all methods did not use covariate data as the trait was not simulated with fixed effects. In the experiments with the trait *y*_2_, because Ott [13] and Anova [16] could not take into account any covariate, we compared our approach with only the maximum likelihood method (ML) [17] with sex, age and race as covariates for fair comparison. The ML software package, downloaded from http://www.genetics.ucla.edu/software/mendel, had the default maximum number of iterations equal to 200, and we also experimented with 500 and 1000. We observed that the ML method could not reach convergence in the experiments with even 20 phenotypic features within a reasonable time limit (two days). Due to this computational hurdle, the ML method could not be applied to datasets with over 20 features.

#### Observations from simulations

**Fig 1:**
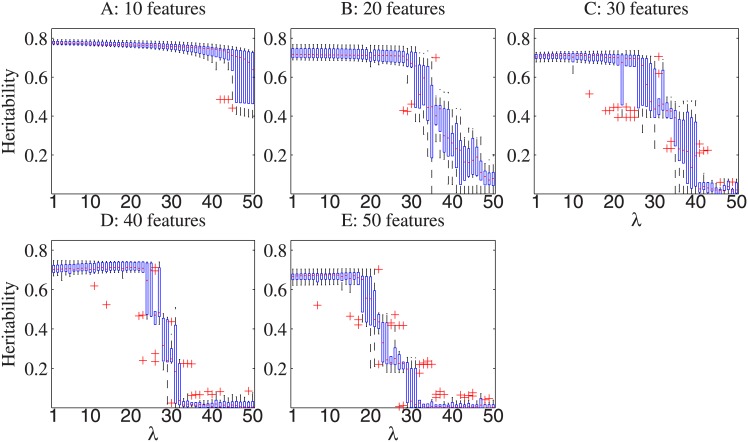
Three-fold cross validated heritability of the linear models derived for the trait *y*_1_ (simulated without covariate effects) when *λ* varies from 0 to 50 with a step size 1, on synthetic datasets consisting of 10, 20, 30, 40 and 50 features.

**Fig 2:**
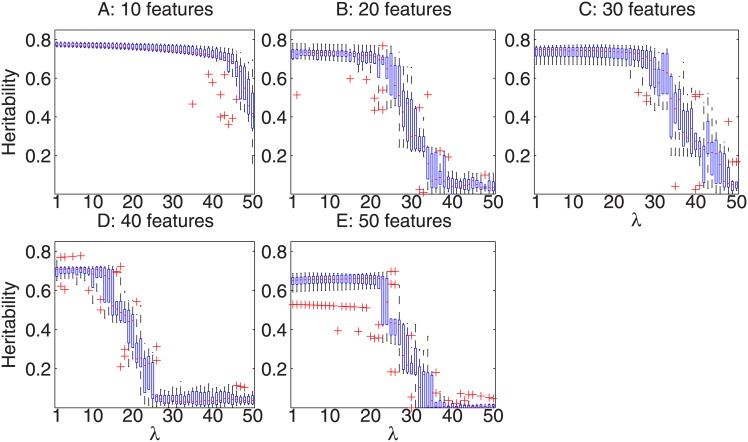
Three-fold cross validated heritability of the linear models derived for the trait *y*_2_ (simulated with covariate effects) when *λ* varies from 0 to 50 with a step size 1, on synthetic datasets consisting of 10, 20, 30, 40 and 50 features.

We first examined the algorithmic behavior of the proposed approach. Fig 1 shows box plots of three-fold cross validated heritability (average values over the 10 trials and standard deviations) of the linear models derived by our approach for the simulated trait *y*_1_ from the five datasets. We observed that the proposed method was able to recover the linearly-combined traits with a relatively wide range of *λ* choices. From Fig 1, when *λ* = 1, 1, 13, 18, and 18 respectively for the five datasets, the best validation heritability was obtained. This observation shows that when the underlying model gets sparse, larger *λ* is favorable to prevent overfitting by removing irrelevant features. We had similar observations in the experiments with *y*_2_ as shown in Fig 2. Fig 2 reports the same box plots for the simulated trait *y*_2_. The validation heritability of the derived traits were high (with a small decrease when more irrelevant features were experimented), which demonstrated that the proposed approach could effectively correct for covariates in finding heritable components.

We next examined the comparison of our approach against the state of the art. To be more thorough, we compared all four methods using four different metrics including validated heritablity, sum of squared residuals to the simulated trait *y*_1_ or *y*_2_ (SE(trait)), squared difference between the learned weights $\hat{w}$ and the true weights **w**, i.e., $||w-\hat{w}{\left|\right|}^{2}$ (SE(**w**)), as well as the computation cost. Table 2 shows the cross validated heritability of the traits derived by each of the methods in the two sets of experiments with *y*_1_ and *y*_2_. The performance was reported with the best *λ* choice of each method. It is clear that the traits derived by our approach always achieved the highest heritability.

**Table 2:** Cross validated heritability of the traits derived by different methods in the experiments without covariates (results are presented in rows from 2 to 5) and with covariates (results are presented in rows 6 and 7).

Method	10 features	20 features	30 features	40 features	50 features
Proposed	**0.777**(0.009)	**0.724**(0.027)	**0.707**(0.018)	**0.717**(0.021)	**0.670**(0.024)
Anova	0.638(0.063)	0.581(0.043)	0.430(0.042)	0.551(0.050)	0.447(0.060)
Ott	0.378(0.049)	0.465(0.080)	0.292(0.048)	0.398(0.036)	0.352(0.065)
ML	0.755(0.020)	0.046(0.032)	–	–	–
Proposed	0.775(0.010)	0.735(0.023)	0.738(0.030)	0.708(0.031)	0.644(0.051)
ML	0.708(0.097)	0.044(0.037)	–	–	–

The “–” sign indicates that those experiments were infeasible due to prohibitive computation cost.

Table 3 compares the values of SE(trait), SE(**w**), and the computation time in seconds. In particular, the computation cost was measured by running each of the methods on the full datasets when the best *λ* value was used. Across all the datasets, our approach obtained the smallest errors as measured by SE(trait) and SE(**w**). Because Anova used analytic formula to compute covariance matrices, and Ott used a single locus in the covariance estimation, both methods required slightly less computation cost than our approach. However, they were limited only to the situations that had no confounding factors (covariates or other loci) in the heritability calculation. Between the two comprehensive methods, our approach was significantly more efficient than the ML method in computation, making the heritable component analysis with a large number of phenotypic features feasible.

**Table 3:** Comparison of the methods on the sum of squared residuals (SE(trait)), squared difference of the true weights and the learned weights (SE(w)), and the computation time (in seconds) in the experiments without covariates (results are presented in rows from 3 to 7) and with covariates (results are presented in rows from 8 to 12).

Dataset	SE(trait)				SE(**w**)				Computation Time (sec.)			
	Proposed	Anova	Ott	ML	Proposed	Anova	Ott	ML	Proposed	Anova	Ott	ML
10 features	**10.89**	59.03	67.44	57.97	**0.09**	1.35	1.38	1.34	0.61	0.17	0.11	8.24e+02
20 features	**16.62**	60.83	63.08	128.01	**0.17**	1.37	1.39	2.54	0.85	0.19	0.15	1.16e+04
30 features	**19.69**	63.03	72.46	–	**0.21**	1.38	1.48	–	0.90	0.19	0.14	–
40 features	**23.31**	62.71	68.39	–	**0.27**	1.39	1.44	–	0.98	0.29	0.23	–
50 features	**25.23**	64.22	67.23	–	**0.29**	1.40	1.43	–	2.13	0.30	0.26	–
10 features	**13.61**	*	*	85.98	**0.11**	*	*	1.35	0.86	*	*	8.85e+02
20 features	**16.14**	*	*	173.40	**0.18**	*	*	2.58	1.07	*	*	1.20e+04
30 features	**26.60**	*	*	–	**0.31**	*	*	–	1.30	*	*	–
40 features	**26.81**	*	*	–	**0.29**	*	*	–	1.61	*	*	–
50 features	**25.87**	*	*	–	**0.31**	*	*	–	2.52	*	*	–

The “–” sign indicates that those experiments were infeasible due to prohibitive computation cost. The “*” sign indicates that the corresponding methods were not tested due to the limitation of the methods that could not handle covariates. The computation time reported for the ML method was measured when the maximum number of iterations was set to 200.

**Fig 3:**
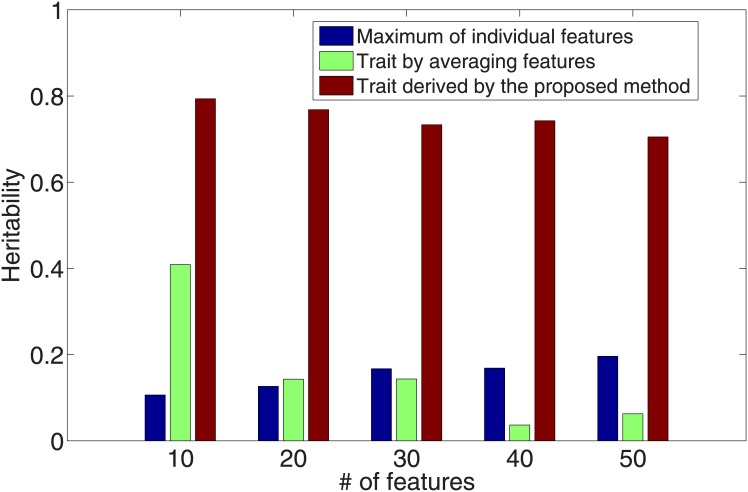
Heritability comparison between the trait derived by the proposed approach, individual features and the simple average of features (without covariate effects).

**Fig 4:**
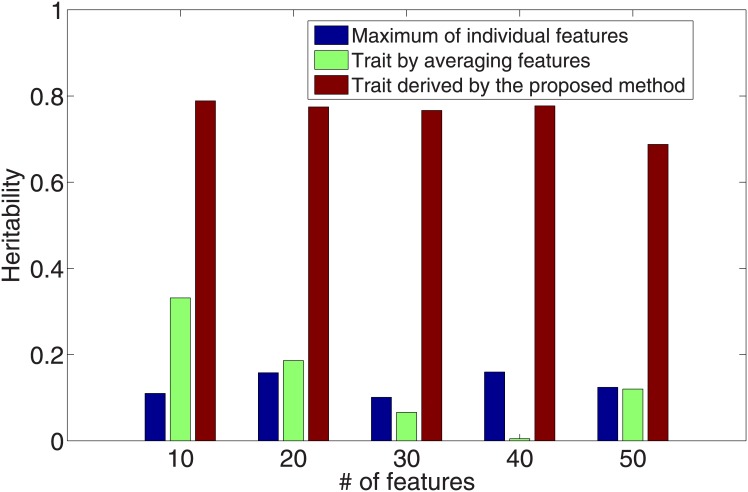
Heritability comparison between the trait derived by the proposed approach, individual features and the simple average of features (with covariate effects).

Our approach identified multivariate traits of much higher heritability than the commonly used traits. We compared the heritability of the traits derived by our approach against that of commonly-used features. We used the traits derived by our approach from the cross validation process when the best *λ* values were used. As shown in Fig 3 (without covariates) and Fig 4 (with covariates), the validation heritability of the derived traits were significantly higher than that of individual features and the average of them.

**Fig 5:**
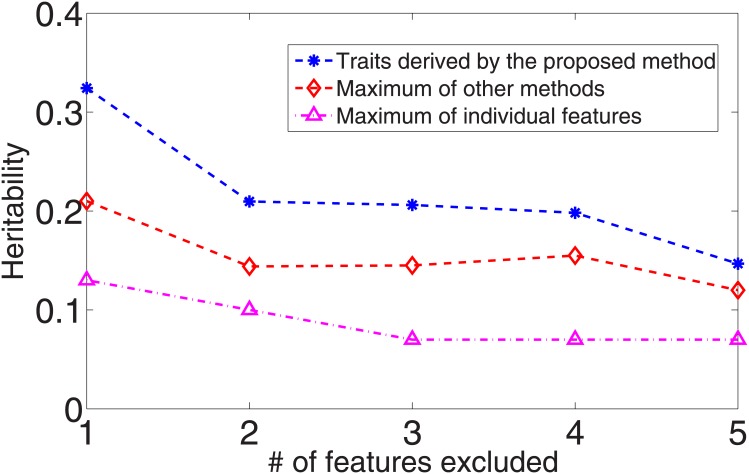
Heritability comparison between the traits derived by the proposed approach, by other methods, and original features when relevant features were randomly selected and excluded from the training data.

Without loss of generality, we used the 20 feature dataset that we synthesized for *y*_1_ to evaluate if our approach could still find heritable components when the groundtruth models were broken. The results are reported in Fig 5 where we compared the heritability of our derived traits against the maximum heritability that other methods could reach and that of the original features. Clearly, the traits derived by our approach achieved much higher heritability.

### A Case Study: Cocaine Use and Related Behaviors

We applied the proposed approach to a genetic study of cocaine use and related behaviors. Two independent sets of samples were used in our analysis: the SSADDA dataset [7], which was used for discovery; and the *Study of Addiction: Genetics and Environment* (SAGE) dataset [25], which was used for replication of the SSADDA findings. The SAGE data were aggregated from multiple NIH-funded projects [26] by NIH’s dbGap system. We downloaded the data from the dbGap public domain [25] through dbGap accession number phs000092.v1.p.

The SSADDA sample included 4895 unrelated individuals which were used in our analysis to help estimate the total phenotypic variance even though they had no effect on the covariance estimates. The SAGE dataset consisted of 58 individuals from nuclear families and 1603 unrelated individuals. The two datasets contained samples from two populations: African American (AA) and European American (EA).

All subjects were reported to have used cocaine in their lifetime, and were assessed on the following 13 features of cocaine use and related behaviors:

- *F1*—tolerance to cocaine;
- *F2*—withdrawal from cocaine;
- *F3*—using cocaine in larger amounts or over longer period than intended;
- *F4*—persistent desire or unsuccessful efforts to cut down or control cocaine use;
- *F5*—great amount of time spent in activities necessary to obtain, use or recover from the effects of cocaine;
- *F6*—gave up or reduced important social, occupational, or recreational activities because of cocaine use;
- *F7*—cocaine use despite knowledge of persistent or recurrent physical or psychological problems likely to have been caused or exacerbated by cocaine;
- *F8*—number of cocaine symptom endorsed;
- *F9*—age when first used cocaine;
- *F10*—age when last used cocaine;
- *F11*—age when first being diagnosed with DSM4 cocaine dependence;
- *F12*—age when last being diagnosed with DSM4 cocaine dependence;
- *F13*—transition time in years between the first time cocaine use and the first cocaine dependence diagnosis.

Features *F1–F7* were binary variables that took a value of “yes = 1” or “no = 0”, and *F8–F13* were continuous variables, which we normalized to the range of [0, 1] in the analysis.

The majority of the 6810 subjects interviewed with the SSADDA, were genotyped on an Illumina microarray for 988,306 autosomal single-nucleotide polymorphisms (SNPs). Genotypes for additional 37,427,733 SNPs were imputed using IMPUTE2 [27] from genotyped SNPs and 1000 Genomes reference panel released in June 2011 (http://www.1000genomes.org). Both subjects and SNPs were undergone stringent quality control (readers can consult with [7] for details). After data cleaning, there were a total of 4,845 subjects (2674 AAs, 2171 EAs) and 30,078,279 SNPs (695,308 genotyped) remained for analysis. Top three ancestral principal components were computed using 145,472 SNPs that were common to discovery samples and the Hapmap panel. All of the 1661 SAGE subjects (640 AAs, 1021 EAs) in the replication dataset were genotyped for 1,072,657 SNPs.

We derived a multivariate trait based on the 13 features of cocaine use and related behaviors. This trait was derived from the SSADDA data by Algorithm 1 with a correction for the fixed effects of age and race. Three-fold cross validation was performed to find the optimal *λ*, which was subsequently used to find a linearly combined trait from the 13 features based on the entire SSADDA data. The heritability of the derived trait was estimated and compared to that of individual quantitative features in the data, including the cocaine symptom count (*F8*). The feature *F8* was recognized as a better trait than the binary trait induced by the diagnosis of cocaine dependence in a recent genomewide association study (GWAS) [7]. We compared the utility of the derived trait and the symptom count as traits in an association analysis. Association tests were performed on the SSADDA sample for both traits and separately for EAs and AAs to identify significant genetic markers at *p* < 5 × 10^−6^. We then computed the derived trait for the subjects in the replication SAGE sample. The markers identified from the SSADDA data were tested using the replication subjects. All tests included age, sex and the first three ancestral principal components as covariates. The association test results on discovery and replication data were combined by performing meta analysis using Metal [28]. Genomewide associations were identified from the meta analysis. Note that the heritability of the derived trait was not estimated on the SAGE data because 97% of the SAGE subjects were unrelated individuals.

**Fig 6:**
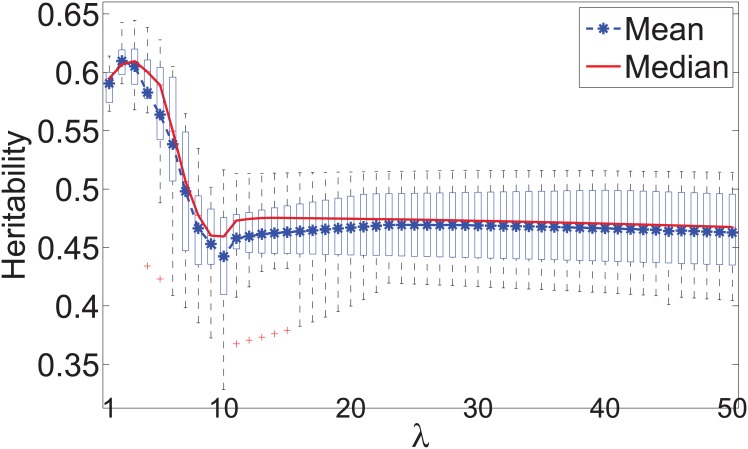
Validation heritability of the multivariate traits derived by our approach for cocaine use and related behaviors using different values of *λ*.

Fig 6 shows the box plots of the cross validated heritability of the traits derived by Algorithm 1 when *λ* varied from 1 to 50 with a step size 1. When *λ* = 2, we observed the highest heritability on average in the cross validation. We hence used *λ* = 2 in Algorithm 1, and derived a linear combination of the features from the entire SSADDA data. The heritability of the derived trait and all individual quantitative features was estimated using SOLAR and reported in Table 4. The quantitative trait derived by our approach has substantially higher heritability than that of all other traits.

**Table 4:** Heritability estimates for the multivariate trait derived by the proposed method and all individual quantitative features in the data.

Traits	heritability	*p*-value	standard deviation
Trait derived by proposed method	**0.70**	4.36 × 10^−22^	0.06
Cocaine symptom count	0.41	1.52 × 10^−08^	0.07
Age when first used cocaine	0.39	2.41 × 10^−09^	0.07
Age when last used cocaine	0.35	6.70 × 10^−06^	0.10
Age when first CD diagnosis	0.43	1.15 × 10^−10^	0.07
Age when last CD diagnosis	0.38	5.99 × 10^−09^	0.07
Transition time between first cocaine use and CD diagnosis	0.42	8.09 × 10^−10^	0.07

**Fig 7:**
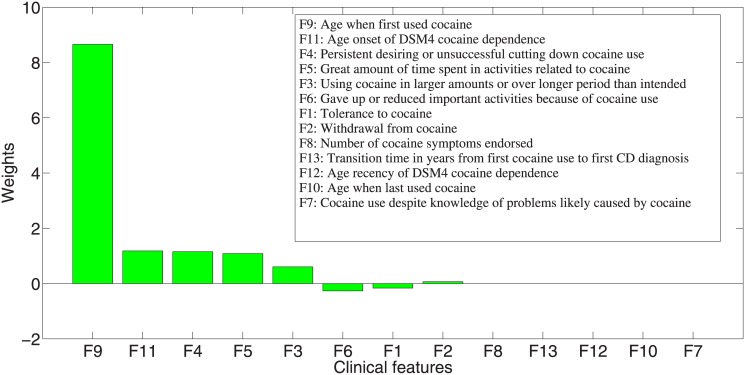
Weights of the eight clinical features in the linear model of the composite trait derived by our approach to the evaluation of cocaine use and related behaviors.

Using a regularization condition based on the sparsity-favoring ℓ_1_ vector norm created shrinkage effects on our model. In other words, our approach selected parsimonious features to use in the linear combination. Fig 7 shows the combination weights of the features obtained in our model. Five of the 13 features had weight of 0, thus were not used by the model. The feature—*age when first used cocaine* received the largest positive weight and therefore had the strongest impact on the derived trait. The other four important features were *F11*—*age onset of DSM4 CD diagnosis*, *F4*—*persistent desire or unsuccessful efforts to cut down or control cocaine use*, *F5*—*great amount of time spent in activities necessary to obtain, use or recover from the effects of cocaine*, and *F3*—*using cocaine in larger amounts or over longer period than intended*. Features *F6*, *F1* and *F2* had some but limited effect on the derived trait.

We identified three SNPs for the AA population and four SNPs for the EA population that passed our *p*-value threshold (5 × 10^−6^) in the genomewide association tests with the discovery sample. These SNPs are listed in Table 5. In recent GWAS of substance use disorders, meta analysis was commonly used to identify genomewide significant associations, e.g., [7, 23, 29]. Following the same strategy in [7], we identified significant markers from the meta analysis results. Another recent study that used the same 1000 Genomes reference panel identified that 10^−8^ is an appropriate p-value threshold for use in a GWAS that employs imputed SNPs [30]. Based on this threshold, the markers rs833936 and rs7224135 in Table 5 were significantly associated with the derived trait at the genomewide level, respectively for AAs and EAs, but not with the commonly-used cocaine symptom count. The other five markers in Table 5 were nominally significantly (1 × 10^−8^< meta *p*-value < 5 × 10^−6^) associated with the derived trait only. In other words, using the standard phenotype in association tests would not discover these SNPs that are associated with a specific subtype (a quantitative subphenotype) of cocaine dependence. The marker rs833936 is located at the *TXNIP* gene which may act as an oxidative stress mediator when its expression is suppressed by synaptic activity in brain [31]. Two markers rs11079045 and rs7224135 are located at the *PTRF* gene which has been identified to be associated with cocaine abuse in an early transcriptional change study [32]. The *EFEMP1* gene has not been reported in the genetic analysis of cocaine dependence. Since all the identified SNP markers have not been thoroughly studied in genetics of cocaine dependence, our findings may promote subsequent investigations for these genes as well as subtypes of cocaine dependence. The proposed heritable component analysis for multivariate phenotypes may provide a new strategy to improve genomewide association studies of complex disorders.

**Table 5:** Top findings obtained by the genome-wide association analysis with the derived subphenotype.

	SNP	Chr	Gene	Discovery			Replication			Meta		
				MAF	*p*_*derived*_	*p*_*symp*_	MAF	*p*_*derived*_	*p*_*symp*_	*p*_*derived*_	*p*_*symp*_
AA	rs769065	6	*DNAH8*	0.26	6.14 × 10^−6^	9.62 × 10^−2^	0.03	8.74 × 10^−3^	3.58 × 10^−2^	1.85 × 10^−7^	1.57 × 10^−2^
	**rs833936**	1	*TXNIP*	0.36	7.90 × 10^−8^	2.51 × 10^−2^	0.12	2.22 × 10^−2^	1.76 × 10^−2^	**5.59** **×** **10** ^**−9**^	2.43 × 10^−3^
	rs75621732	11	*MLSTD2*	0.06	1.89 × 10^−6^	1.85 × 10^−1^	0.35	4.95 × 10^−2^	5.60 × 10^−1^	2.70 × 10^−7^	1.48 × 10^−1^
EA	rs11079045	17	*PTRF*	0.40	2.48 × 10^−6^	2.24 × 10^−1^	0.42	1.48 × 10^−3^	2.24 × 10^−1^	1.33 × 10^−8^	1.82 × 10^−1^
	**rs7224135**	17	*PTRF*	0.40	7.61 × 10^−7^	1.50 × 10^−1^	0.41	2.29 × 10^−3^	1.50 × 10^−1^	**6.51** **×** **10** ^**−9**^	1.08 × 10^−1^
	rs10490394	2	*EFEMP1*	0.20	8.78 × 10^−7^	1.53 × 10^−1^	0.19	9.15 × 10^−3^	1.53 × 10^−1^	3.22 × 10^−8^	2.33 × 10^−1^
	rs7330895	13	*DACH1*	0.39	7.50 × 10^−6^	6.00 × 10^−2^	0.34	2.81 × 10^−2^	6.00 × 10^−2^	8.00 × 10^−7^	2.80 × 10^−3^

Notes: Chr—chromosome; MAF—minor allele frequency; *p*_*derived*_—the *p*-value obtained with the trait derived by the proposed method; *p*_*symp*_—the *p*-value obtained with the cocaine symptom count. SNPs with *p*-values that reach genome-wide significant level (< 10^−8^) are in bold font.

## Discussion and Conclusion

In this paper, we have proposed a quadratic optimization formulation that is capable of identifying highly heritable components of complex phenotypes. The multivariate trait is derived as a linear function *y* = **x** ^⊤^ **w** of lower level traits **x** by explicitly maximizing its heritability. Specifically, we search for the optimal **w** that maximizes the likelihood of observing a high value of heritability. This is equivalent to finding the best **w**, so that the projected trait **x** ^⊤^ **w** will be best aligned with the kinship matrix **Φ** of the pedigree. An efficient algorithm based on sequential quadratic programming has been developed to optimize the proposed formulation. The algorithm is extended to allow the correction for covariate effects when deriving a heritable component.

Our simulation study provides evidence of the effectiveness of the proposed approach as a means to find highly heritable components of multivariate phenotypes. Then a case study on the phenotypes of cocaine use and dependence was conducted. A quantitative trait was identified based on thirteen cocaine use symptoms and behaviors. The trait had a heritability estimate of 0.7 (with *p* = 4.36 × 10^−22^, std = 0.06), which was much higher than a standard cocaine-use phenotype, e.g., the symptom-count trait, with heritability of 0.41. The subsequent phenotype-genotype association study demonstrated important utility of the derived trait for use in association analysis. Our results show that seven SNPs were significantly or nominally significantly associated with the derived subphenotype, but were not associated with the symptom count phenotype. Two out of the seven associated SNPs reached genome-wide significant level after correction for multi-testing following the procedure in [7, 30].

Our formulation has a hyper-parameter *λ*. Using a hyper-parameter is common in machine learning algorithms such as support vector machines [33]. As a hyper-parameter, *λ* is not determined by solving the formulation itself and instead needs to be pre-specified. Both our simulation study and our case study showed that our formulation is fairly robust to the value of *λ* when it is chosen from a reasonably wide range. In real-world applications, hyper-parameters are often determined by a cross-validation process, which was used in our experiments.

Discovering heritable components of a multivariate phenotype can also improve genomic prediction [34]. If a trait is highly heritable, a model that is based on genomic markers to predict the trait value can achieve high accuracy [35]. In agricultural science, heritability of the breeding trait is considered to be one of the most important factors for the performance of a breeding program. Breeding programs targeted at conceptual but economically important phenotypes, such as feed efficiency or heat tolerance of animals, are confronted with a wide variety of available measures for the phenotype [36, 37]. Residual body weight gain, residual feed intake, or relative growth rate are feed efficiency measures for dairy cattle with heritability ranging from 0.28 to 0.45 [36, 38]. Each of these measures forms a multivariate trait that is defined by a linear function of low level traits, such as body weight, diet and feed energy intake, and days in milk. Our new algorithm can help the identification of more heritable measures for conceptual phenotypes of animal or plant.

There are limitations of our proposed technique. The non-convex quadratic optimization formulation requires a complex solver, such as sequential quadratic programming. For a sample that contains millions of subjects, it may become computationally prohibitive. More efficient solvers or approximations may be needed to scale up the proposed approach. In some applications, complex grouping structures may exist in the data between different lower level traits. A formulation that takes into account the special data structure may be more useful in producing biologically and clinically meaningful traits. As discussed in the paper, alternative regularization conditions exist, including some that may deal well with complex data structures, such as the one based on ℓ_2,1_ vector norm. Algorithms that can solve the formulations with alternative regularization terms need to be developed. Additional empirical studies across different disciplines are needed to evaluate the capability and effectiveness of the proposed approach.
